# Neuromuscular, Perceptual, and Temporal Determinants of Movement Patterns in Wheelchair Fencing: Preliminary Study

**DOI:** 10.1155/2020/6584832

**Published:** 2020-05-04

**Authors:** Zbigniew Borysiuk, Tadeusz Nowicki, Katarzyna Piechota, Monika Błaszczyszyn

**Affiliations:** ^1^Faculty of Physical Education and Physiotherapy, Opole University of Technology, 45-758 Opole, Prószkowska 76, Poland; ^2^Sport Club for Integration at the Academy of Physical Education in Warsaw, Poland

## Abstract

The objective of the present study was to determine the structure of the movement pattern performed during a wheelchair fencing lunge that is executed in response to visual and sensory stimuli. In addition, a comparison was made between fencers in the categories A and B of disability. In addition, the analysis involved the correlation between the duration of the sensorimotor response and the value of the bioelectric signal recorded in selected muscles. Seven Paralympic team athletes specializing in wheelchair fencing (3 in category A and 4 in category B) participated in the research. The fencers perform at international level competitions and are multiple medalists of the Paralympic Games. In the study, a wireless system for sEMG and accelerometer signal measurement was employed to test the intervals between the initiation of the lunge attack and its termination defined by the touch of the weapon on the coach's torso. The electrodes were placed on 9 key muscles responsible for the effectiveness of the executed attack: DEL, TRI, BC, ECR FCR, LD, and EAO. The significant intergroup difference in the muscle activation was found to be 0.333 s for category A fencers and 0.522 s for category A fencers at *p* = 0.039 applies to the *latissimus dorsi* (LD LT) muscle, which demonstrates its significance as a postural muscle in the structure of the examined movement pattern. In terms of the values of EMG, a tendency for higher MVC (%) values in most muscles for category A competitors was recorded. The *latissimus dorsi* (DL RT) muscle with an intergroup difference of MVC-114.63 for cat. A and 67.50 for cat. B at *p* = 0.039 turned out to play a significant role. The results prove the role of postural muscles: *external abdominal oblique* and *latissimus dorsi* on the effectiveness of the attacks executed in wheelchair fencing.

## 1. Introduction

Wheelchair fencing dates back to 1948, when Dr. Ludwik Gutman included it in the rehabilitation programs of veterans who recovered after World War II. It took place at the Rehabilitation Center for disabled soldiers at Stoke Mandeville Hospital in Buckinghamshire, UK. Since 1960, wheelchair fencing has been present in the Paralympic Games in Rome [1]. In 2006, integrated tournaments were conducted at the Turin World Cup and for both able-bodied fencers and ones with disabilities. Practicing fencing in wheelchairs offers a considerable recreational and health-related value and provides an excellent rehabilitation procedure for people on wheelchairs. Nowadays, however, after it has been practiced for over half a century at Paralympics and international tournaments, this discipline has assumed the status of competitive sport, and in this context, it is considered in scientific research.

The research concerned with wheelchair fencing conducted to this date has focused on the issues relating to injuries that wheelchair fencers are subjected to [2–5]. A significant proportion of the research involves physiological and conditioning determinants of achievement potential in this sport [6] and various aspects of kinematics of movement patterns performed by disabled fencers [7]. Many works deal with biomechanical analysis that applies motion capture and EMG systems [8] as well as comparisons between competitors who have trained wheelchair fencing and the ones that practice other sport disciplines [9]. Due to the important role taken on by the introduction of sport specific classification systems and the identification of specific categories, researchers refer to these issues as the objectivity of the adopted criteria [10, 11].

There are three divisions or categories of competition, with divisions based upon the categories of disability. The fencers are classified into one of three categories: A, B, or C. Athletes classified as an “A” have more ability, e.g., such athletes are amputees or were diagnosed with mild paralysis within the lower limbs, but they are free to perform movements in the regions of their torso and arms. Category B athletes have spinal cord injuries and paresis of legs and partial arms (paraplegic), while category C includes the athletes with most serious forms of disability with four-limb paralysis (tetraplegic). However, only athletes classify as category A and B took part in this study. Due to the fact that the study applied athletes who use thrusting weapons who are trained épée and foil, during the tests, the players used an épée with a weight of 750 g. The surface hit by the weapon includes the torso and arms and a head mask. Wheelchair fencing forms a direct derivative of fencing practiced by nondisabled athletes. The regulations, system of point award, as well as competition rules are identical. In training, the basis is individual lessons, and so-called duels form the basis of the preparation of the competitors [12, 13]. The fencers with disabilities are characterized by special dynamic characteristics, which involve a lot of muscles, primarily ones in the region of the arms and forearms, as well as the trunk on the back and stomach. Wheelchair fencing is defined as a psychomotor sport in which coordination-related abilities (reaction times, movements times, kinesthetic sensation, concentration, and focus of attention) need to be harmonized with strength and explosiveness as exercise capacity.

The design of the wheelchairs provides extraordinary dynamics of movement of the torso together with an arm holding the weapon, which determines the fencers' range of attack.

Numerous studies of fencers without disabilities have proved the role of the legs in the structure of movement patterns that also perform important postural functions. With regard to wheelchair fencing, an important role is taken on by selected muscles in the region of the abdomen and torso [7].

The research applied surface electromyography (sEMG) and accelerometer techniques as the main research tool, and the study was based on the following assumptions:

1. the structure of the movement pattern (the order of bioelectric muscle activation) during the lunge attack on the coach's torso in both category A and B competitors is marked by the regularity involving the initiation of the pattern by the extensor muscle of the arm (TRI RT) followed by the activation of the abdominal and back muscles as the muscles responsible for postural functions [14, 15]
2. in terms of the complex response times (CRT), movement time from the execution of the thrust by the trainer until the touch of the blade on the trainer's torso by the player, and category A fencers demonstrate the best results
3. a similar tendency in terms of CRT values in response to the kinesthetic stimulus is assumed both in the category A and B fencers
4. higher EMG values (MVC (%)) should be recorded in fencers representing category A in the key muscles to represent increased movement dynamics associated with the activation of additional motor units
5. a significant relation is forecasted to be established between MVC (%) parameters of selected muscles and the value of CRT

## 2. Methodology

### 2.1. Subjects

7 competitors of the Paralympic team of wheelchair fencing (3 from category A and 4 from category B) took part in the present study. Selected fencers represent a high international level. The subjects include multiple medal winners of the Paralympic Games.  Table 1 presents the basic date of the regarding fencers in categories A and B.

The scope of the study was accepted by the decision of the Bioethics Committee of the Medical Chamber (Resolution No. 237 of 13 December 2016) regarding the guidelines described in the Helsinki Declaration regarding the conduct of clinical trials in humans.

### 2.2. Procedures

The tests were performed using a 16-channel EMG system (Noraxon, DTS, Desktop Direct Transmission System, Scottsdale, ZA, USA) with a sampling frequency of 16 bits for a resolution of 1500 Hz. Dedicated software was applied for the analysis of the system data (MyoResearch XP Master Edition for DTS Noraxon). A wireless transmitter-recorder was used to synchronize the EMG system and transfer the EMG signal directly to the computer system (3-axis wireless DTS 3D accelerometer sensor with following specifications: nominal output range: +/- 6 g, sensitivity +/- 0.67 V/g, and bandwidth 5 Hz–1.8 kHz).

The entire research procedure was carried out in accordance with the principles of the SENIAM project. Among the 3 attempts performed in the series of the activities, attempt no. 2 was most often subjected to detailed analysis.

The sEMG signals were smoothed by estimating the square root mean in a window of 300 ms.

One of the EMG indicators involved the determination of a maximum value. This was also performed by the application of the MyoResearch XP Mater Edition software. The maximum EMG value was obtained based on the application of data normalization of 3 trials. The MVC reference value was calculated in a time window of 50 ms for which the mean value of the sEMG signal was the highest. All signals were normalized in reference to these values and expressed in percent.

The order of bioelectric muscle activation was determined on the basis of the baseline determined by the peak values of selected muscle groups. The MyoResearch XP Mater Edition software was employed for the determination of the baseline threshold to establish the instant corresponding to the activation (Onset/Offset) of the muscles. The method applied to estimate Onset and Offset thresholds involved the determination of the local peak = 5%.

The next analyzed indicator involved the use of Complex Response Time (CRT) calculated as the interval between the first stepwise acceleration of the signal and the highest value of EMG.

### 2.3. Methods

The experimental procedure was preceded by an individual warm-up training session with a coach lasting for 20–25 minutes. After a typical technical session with the coach, the fencers were prepared for the test procedure. In the study, the electrodes were placed on 9 spots on the bodies of the subjects: on arm muscles (DEL RT, TRI RT, and BC RT), on forearm muscles (ECR RT, FCR RT) as well as on abdominal and dorsal muscles (EAO RT and LT, LD RT and LT). The list with the muscle names is given below.

List of muscles and their names:

RT: right side
LT: left side
DEL: Deltoideus middle head
TRI: Triceps brachii
BC: Biceps brachii
ECR: Extensor carpi radialis longus
FCR: Flexor carpi radialis
LD: Latissimus dorsi
EAO: External abdominal oblique
x, y, z channel: accelerometer in 3 axes

Before the initiation of the trial tests, the fencing strip with the fencers' wheelchairs was set up so that the end of the weapon was located at adjusted position from the bent arm of the coach (photo 1). Throughout the test procedure, the coach initiated three series of simple lunge attacks in response to a visual stimulus (at a signal given by the coach's blade from the parry quarte to parry sixte and in response to a sensory stimulus—as the fencers executed an attack at the instant when the coach's weapon was detached from the one held by the fencer).

**Figure 1:**
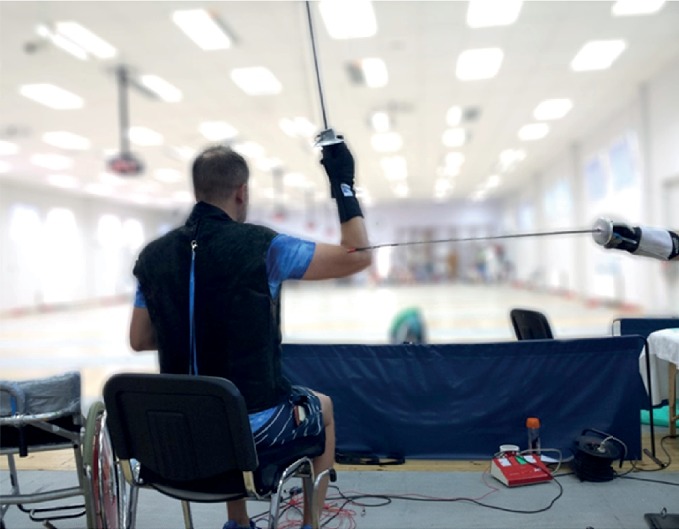
Adjustment of fencing piste—distance between the tip of the fencer's weapon and coach's elbow and accelerometer located on the guard of the coach's weapon.

**Figure 2:**
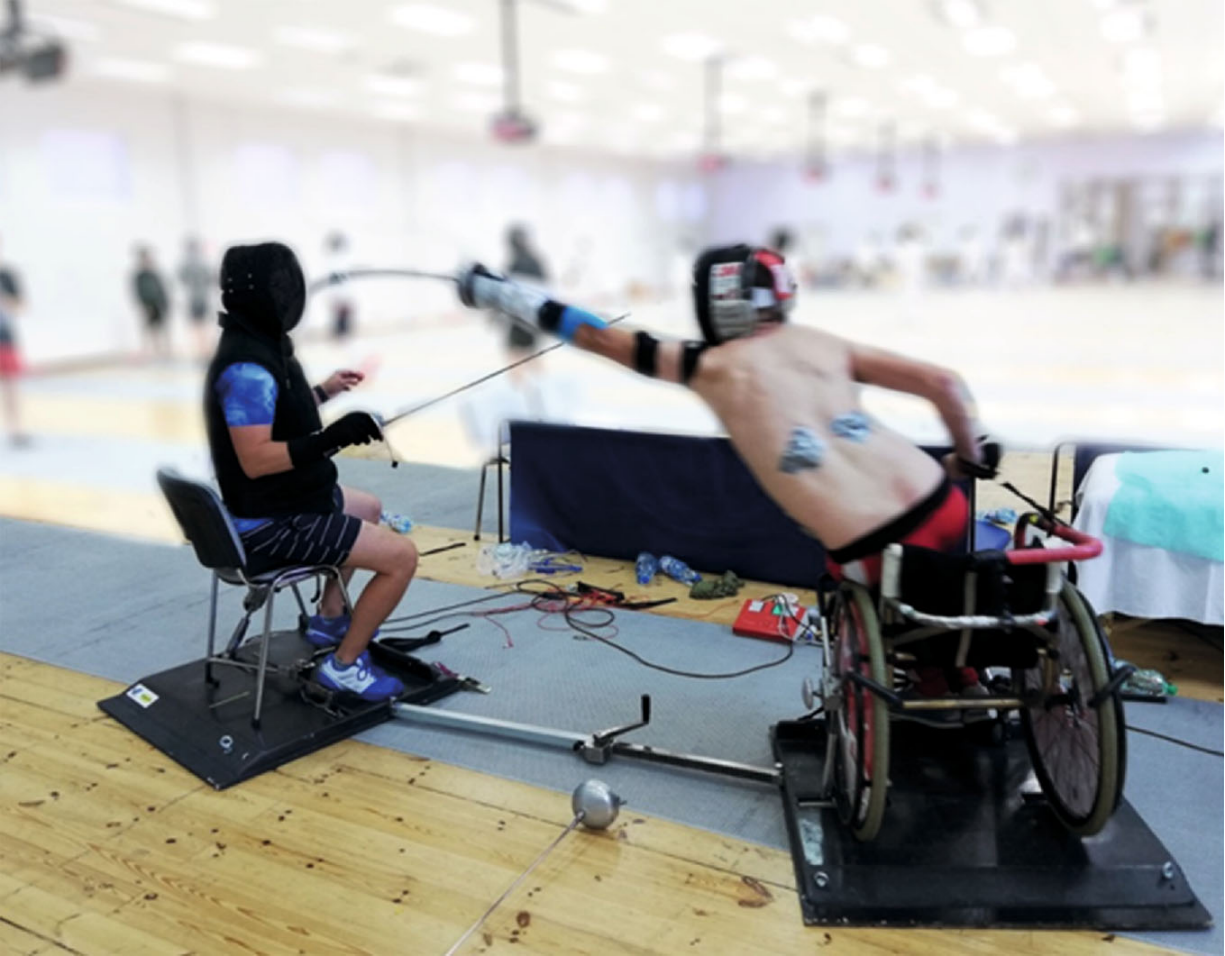
A lunge executed on the coach's torso.

Figure 1 shows the standard distance between the end of a fencer's weapon and the coach's elbow. In addition, an accelerometer was placed on the guard of the coach's weapon.  Figure 2 presents a lunge executed on the coach's torso and locations of electrodes attached to the subjects' bodies.

### 2.4. Statistical Analysis

The material gained from the research was developed using Statistica 13.1 (StatSoft, Inc., OK, USA). All the hypotheses considered in the paper were verified at significance level *p* ≤ 0.05. The assumption about the normal distribution of the analyzed statistical features was tested using the Shapiro-Wilk test.

Due to the fact that not all the features have met the assumptions of normal distribution, to test the interdependence of its nonparametric tool—Spearman rank-order correlation (R) and the nonparametric Wald-Wolfowitz Runs Test were applied for this purpose.

## 3. Results

**Figure 3:**
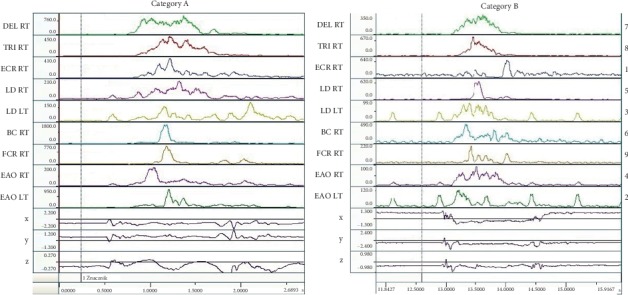
Charts representing the order of the activation of the muscles in response to a visual stimulus in category A and B fencers.

Figure 3 presents the order of muscle activation in response to a visual stimulus. On its basis, it can be concluded that subjects in category A, the muscles in the torso, and in the back were the first to generate an electrical signal, that is *latissimus dorsi* (flexion of LD RT) and LD LT, followed by the activation of the *deltoideus middle head* (DEL RT). Subsequently, *external abdominal oblique* (EAO RT) and *extensor carpi radialis longus* (ECR RT) and *triceps brachii* (TRI RT) generated an electric signal. At the end of the attack executed on the coach's torso, the flexors BC RT and FCR RT were activated.

In contrast, in the fencers in the category B (Figure 3), the analysis found that the first stimulation in response to a visual stimulus was initiated by the activation of the *extensor carpi radialis longus* (ECR RT), followed by *external abdominal oblique* (EAO LT and RT) and *latissimus dorsi* (LD LT and RT), and followed by the arm muscles (BC RT, DEL RT, and TRI RT). At the end of the attack executed on the coach's torso, the *flexor carpi radialis* (FCR RT) produced signal meaning its activation.

On the basis of the analysis of Table 2, we can conclude about the existence of a statistically significant difference between the two examined groups in terms of complex reaction time representing the activation of the muscles: LD LT (*p* = 0.039, *Z* = −2.062).

On the basis of the analysis in Table 3, we can conclude about the existence of a statistically significant relation between the two examined groups in terms of the value MVC (%) of the activation of the muscle DEL LT (*p* = 0.039, *Z* = −2.062).

**Figure 4:**
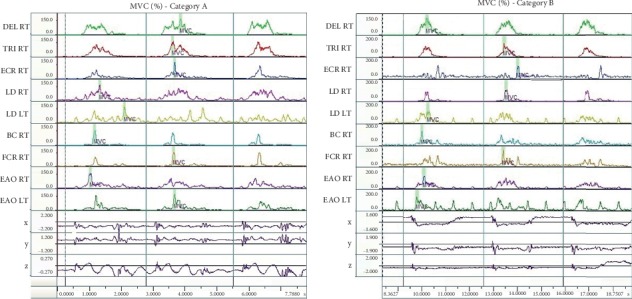
Charts representing MVC (%) values for selected fencers in category A and B—in response to visual stimulus.

Figure 4 demonstrates the course of the EMG value expressed in terms of MVC (%) to give the results of all three tests—performed by a representative fencer in category A and B. The values give the fencers reaction in response to the visual stimulus.

On the basis of the data in Table 4, statistical differences were not established between the fencer categories in terms of complex reaction times in response to a sensory stimulus.

On the basis of the analysis of the data in Table 5, we can conclude about the occurrence of significant differences between the categories of fencers in terms of the value MVC (%) representing activation of TRI RT muscle (*p* = 0.039, *Z* = −2.062).

**Figure 5:**
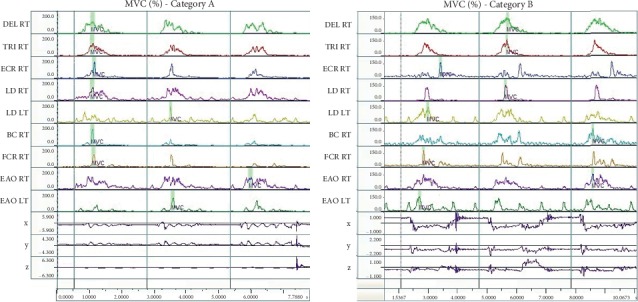
Charts representing the value of EMG (MVC (%)) signal for selected fencers in category A and B—in response to visual stimulus.

Figure 5 presents the course of the value of EMG expressed as MVC (%) to illustrate the results in all three attempts—performed by representative fencers in categories A and B. The presented values apply to the reactions in response to a sensory stimulus.

Taking into account the response to visual stimulus in the fencers categories A and B (*n* = 7), the results of the analysis demonstrate a high correlation coefficient (*r* = −0.821) only between the Complex Reaction Time (CRT) and the value of MVC in the EAO RT muscle.

Also in response to the sensory stimulus, statistical significance (*r* = 0.857) could only be established between CRT and MVC (%), and this applied only to the LD RT muscle. In connection with the above data, the conclusion is that postural muscles, *external abdominal oblique* and *latissimus dorsi*, were the muscles that correlated to the greatest extent with the decrease of the complex motor reaction times.

## 4. Conclusions

The results of the present study have shown that wheelchair fencers are characterized by a specific complex reaction pattern. In terms of the structure of the lunge executed on the coach's torso in response to a visual stimulus, the fencers in category A initiated the patterns by activating the dorsal muscles, then the *deltoid muscle* (DEL) was stimulated, followed by the abdominal muscles (EAO). The activity of *flexor carpi* (ECR RT) and *triceps brachii* (TRI RT) was initiated later. At the end of the phase of the attack on the coach's torso, the *biceps brachii* (BC) and *flexor carpi radialis* (FCR RT) were activated. With regard to category B fencers, we can see that the movement pattern begins in the *deltoid muscle* and then in the abdominal muscles. The further sequence of activation is similar to that in the fencers in category B. Generally, we can emphasize that the important role in this sequence is taken on by the torso and abdominal muscles that perform postural functions [16]. When we take into account the idea of APA—Anticipatory Postural Adjustment [17]—this order of activation is observed first or in synergy with the extensor muscles of the arm. Such considerable dynamic characteristics of the movement pattern lead to body imbalance. In order to maintain postural stability, the central nervous system triggers an anticipatory mechanism that activates postural muscles. A similar phenomenon is observed in the case of able-bodied fencers. The analogy here applies to the anticipatory activation of muscles (from several dozen to about 150 ms) in the rear leg, in particular, visible in the gastrocnemius muscles throughout a fencing lunge [18]. The APA phenomenon is designed not only to maintain the fencer's postural stability on the piste, but also leads to the rapid displacement of the trunk towards the hit area to follow the arm that executes the attack. The result is a shorter response and movement time in response to an opponent's action [19].

The analysis of the activation of particular muscles in response to a visual stimulus demonstrates a slight decrease in the time of complex sensory-motor response (CRT) in all muscles to the advantage of subjects in group A. However, a significant intergroup difference of 0.333 s was noted with regard to the category A fencers and 0.522 s for category B for the *latissimus dorsi* (LD LT), which indicates its important role as a postural muscle in the entire structure of the examined activity. In response to sensory stimuli, statistically significant differences were observed between groups A and B in the timing of individual muscle activation were not established.

Interesting insights are provided by the analysis of EMG bioelectrical signal expressed in terms of MVC (%). A tendency for higher EMG values in most muscles of category A fencers was recorded in this study; however, the key role can be attributed to LD RT muscle with the intergroup difference of 114.63 for category A fencers and 67.50% MVC for subjects in category B. Different characteristics were established in muscle activation in response to the sensory stimulus, where the role of TRI RT muscle seems to be particularly important, and the extensor muscle of the arm generates unexpectedly significantly higher values of EMG signal expressed by MVC (%) in the subjects in the category B—93.73 compared to fencers in category A—78.59.

In accordance with the results of reports offered in various studies concerned with EMG (MVC (%)) signal analysis, the level of this signal should correlate with the response times, in particular with the movement speed and complex reaction times both in fencers on wheelchairs as well as ones without disabilities [20, 21].

By application of the correlation analysis and taking into account the response to visual stimulus, all the fencers in categories A and B (*n* = 7) demonstrated a high level of correlation coefficient *r* = −0.821 only between the complex reaction time (CRT) and the value of EMG% MVC signal produced by an *external abdominal oblique* (EAO RT). Besides, in terms of the response to the sensory stimulus, a significant correlation of *r* = 0.857 was established between CRT and EMG% MVC, only for *latissimus dorsi* (LD RT). It is significant that the postural muscles, *external abdominal oblique* and *latissimus dorsi*, correlated to the highest extent with the decrease of the complex sensorimotor response.

To sum up, we can state that the movement patterns selected as the research procedure (a simple lunge on the trainer's torso in response to a visual and sensory signal) form an original contribution to the area concerned with the assessment of the fencing technique of wheelchair competitors. Although elements of movement patterns performed by wheelchair fencers were previously evaluated by other studies, the results of this analysis can be applied to state the important role of the pattern described by the sequence of muscle activation and the level of bioelectric signal expressed by EMG. The new approach emphasizes the importance of postural muscles: back and abdomen and the role played by them in achieving an effectiveness of executing fencing attacks [22].

The close observation of wheelchair fencing training demonstrates that the training practice performed by them mainly includes individual lessons with trainers and sparring duels with other team members. In the light of the conducted research, it seems necessary to complete the training process by including postural muscle training in the aspect of strength and explosive power development. The activation of additional groups of muscles should contribute to greater coordination capabilities and consequently enhance the speed of attack.

## Figures and Tables

**Table 1:** Basic data regarding fencers in categories A and B.

Fencer	Age	Height (m)	Mass (kg)	Training experience (years)
Category A				
DP	44	1.82	74	23
NC	32	1.82	70	18
RT	32	1.81	82	7
Mean	36	1.82	75.33	16
SD	6.93	0.01	6.11	8.19

Category B				
JG	52	1.8	69	6
AG	17	1.69	60	3
AC	29	1.67	67	11
KR	34	2.00	73	7
Mean	33	1.79	67.25	6.75
SD	14.54	0.15	5.44	3.30

**Table 2:** Statistical analysis (Wald-Wolfowitz Runs Test) based on the comparison of the mean duration of all attempts performed by the selected muscle groups—in response to visual stimulus in category A and B fencers.

Muscle	Variable	Wald-Wolfowitz Runs Test			
		Mean A time (s)	ean B time (s)	*Z*	*p* value
DEL RT	Time (ms)	0.489	0.540	1.334	0.182
TRI RT	Time (ms)	0.560	0.575	0.485	0.628
ECR RT	Time (ms)	0.380	0.527	1.334	0.182
LD RT	Time (ms)	0.420	0.562	-0.364	0.716
LD LT	Time (ms)	0.333	0.522	-2.062	0.039^∗^
BC RT	Time (ms)	0.547	0.520	0.485	0.628
FCR RT	Time (ms)	0.617	0.631	0.485	0.628
EAO RT	Time (ms)	0.408	0.489	-0.364	0.716
EAO LT	Time (ms)	0.435	0.456	0.485	0.628

^∗^ *p* ≤ 0.05.

**Table 3:** Statistical analysis (Wald-Wolfowitz Runs Test) based on the comparison of the mean values of the EMG (MVC (%)) signal in selected muscle groups—in response to visual stimulus in category A and B fencers.

Muscle	Variable	Wald-Wolfowitz Runs Test			
		Mean A time (s)	Mean B time (s)	*Z*	*p* value
DEL RT	MVC (%)	114.63	67.50	-2.062	0.039^∗^
TRI RT	MVC (%)	71.60	44.95	1.334	0.182
ECR RT	MVC (%)	64.00	73.80	-1.213	0.225
LD RT	MVC (%)	94.37	48.60	-1.213	0.225
LD LT	MVC (%)	96.13	59.18	-0.364	0.716
BC RT	MVC (%)	48.67	38.53	0.485	0.628
FCR RT	MVC (%)	77.60	62.15	-0.364	0.716
EAO RT	MVC (%)	75.40	44.75	-0.364	0.716
EAO LT	MVC (%)	79.13	95.63	1.334	0.182

^∗^ *p* ≤ 0.05.

**Table 4:** Statistical analysis (Wald-Wolfowitz Runs Test) of the mean duration of all 3 attempts for selected muscles—in response to sensory stimulus in category A and B fencers.

Muscle	Variable	Wald-Wolfowitz Runs Test			
		Mean A time (s)	Mean B time (s)	*Z*	*p* value
DEL RT	Time	0.503	0.458	0.485	0.628
TRI RT	Time	0.617	0.561	1.334	0.182
ECR RT	Time	0.480	0.507	0.485	0.628
LD RT	Time	0.516	0.423	-0.364	0.716
LD LT	Time	0.361	0.315	1.334	0.182
BC RT	Time	0.514	0.468	1.334	0.182
FCR RT	Time	0.560	0.453	-0.364	0.716
EAO RT	Time	0.437	0.238	-0.364	0.716
EAO LT	Time	0.304	0.446	0.485	0.628

**Table 5:** Statistical analysis (Wald-Wolfowitz Runs Test) of the mean duration the values of EMG signal for selected muscles—in response to sensory stimulus in category A and B fencers.

Muscle	Variable	Wald-Wolfowitz Runs Test			
		Mean A time (s)	Mean B time (s)	*Z*	*p* value
DEL RT	MVC (%)	84.86	113.75	-0.364	0.716
TRI RT	MVC (%)	78.59	93.73	-2.062	0.039^∗^
ECR RT	MVC (%)	76.89	90.31	0.485	0.628
LD RT	MVC (%)	65.63	57.64	-0.364	0.716
LD LT	MVC (%)	58.09	68.86	-1.213	0.225
BC RT	MVC (%)	63.95	56.41	1.334	0.182
FCR RT	MVC (%)	37.91	42.61	0.485	0.628
EAO RT	MVC (%)	78.23	72.58	1.334	0.182
EAO LT	MVC (%)	99.90	75.64	-0.364	0.716

^∗^ *p* ≤ 0.05.

## Data Availability

The data used to support the findings of this study are included within the article.

## Additional Points

Additional Information. In order to generalize the results achieved, it is suggested to increase the sample in the future research.
